# Immunohistochemistry for detection of avian infectious bronchitis virus strain M41 in the proventriculus and nervous system of experimentally infected chicken embryos

**DOI:** 10.1186/1743-422X-6-15

**Published:** 2009-02-05

**Authors:** Ahmed S Abdel-Moneim, Priscila Zlotowski, Jutta Veits, Günther M Keil, Jens P Teifke

**Affiliations:** 1 Virology Department, Faculty of Veterinary Medicine, Beni-Suef University, Beni-Suef 62511, Egypt; 2 Setor de Patologia Veterinária, Faculdade de Veterinária – UFRGS, Av. Bento Gonçalves, 9090, Porto Alegre, RS, Brasil; 3 Friedrich-Loeffler-Institut (FLI), Federal Research Institute for Animal Health, Südufer 10, 17493 Greifswald-Insel Riems, Germany

## Abstract

**Background:**

Infectious bronchitis virus primarily induces a disease of the respiratory system, different IBV strains may show variable tissue tropisms and also affect the oviduct and the kidneys. Proventriculitis was also associated with some new IBV strains. Aim of this study was to investigate by immunohistochemistry (IHC) the tissue tropism of avian infectious bronchitis virus (IBV) strain M41 in experimentally infected chicken embryos.

**Results:**

To this end chicken embryos were inoculated in the allantoic sac with 10^3 ^EID_50 _of IBV M41 at 10 days of age. At 48, 72, and 120 h postinoculation (PI), embryos and chorioallantoic membranes (CAM) were sampled, fixed, and paraffin-wax embedded. Allantoic fluid was also collected and titrated in chicken embryo kidney cells (CEK). The sensitivity of IHC in detecting IBV antigens in the CAM of inoculated eggs matched the virus reisolation and detection in CEK. Using IHC, antigens of IBV were detected in nasal epithelium, trachea, lung, spleen, myocardial vasculature, liver, gastrointestinal tract, kidney, skin, sclera of the eye, spinal cord, as well as in brain neurons of the inoculated embryos. These results were consistent with virus isolation and denote the wide tissue tropism of IBV M41 in the chicken embryo. Most importantly, we found infection of vasculature and smooth muscle of the proventriculus which has not seen before with IBV strain M41.

**Conclusion:**

IHC can be an additional useful tool for diagnosis of IBV infection in chickens and allows further studies to foster a deeper understanding of the pathogenesis of infections with IBV strains of different virulence. Moreover, these results underline that embryonic tissues in addition to CAM could be also used as possible source to generate IBV antigens for diagnostic purposes.

## Background

Infectious bronchitis virus is the prototype species of the family *Coronaviridae *in the order *Nidovirales*. More than 25 genotypes are distributed worldwide. IBV causes an acute highly contagious viral respiratory disease of chickens which is characterized by respiratory rales, coughing and sneezing [1]. Some IBV strains replicate in the gastrointestinal tract, oviduct, and kidney, and due to their nephropathogenic properties they have the potential to cause severe losses with up to 44% mortality [1,2]. In other cases, infection of the proventriculus leads to 75% to 100% mortality in young birds [3]. Most isolates of IBV replicate well in the developing chicken embryo following inoculation of the allantoic cavity, and high titers of virus can be isolated from the allantoic fluid [4]. Replication of IBV strains M41 and Beaudette *in vitro *is restricted to primary chicken cells and depends on the expression of 2,3-linked sialic acids on the cell surface. Thus, these molecules are supposed to serve as receptor determinants for primary attachment of IBV to host cells [5,6].

The conventional diagnosis of the IBV is based on virus isolation in embryonated eggs, followed by immunological identification of isolates. Since two or three blind passages are often required for successful primary isolation of IBV, this procedure could be tedious and time consuming. Alternatively, IBV may be isolated by inoculation in chicken tracheal organ cultures. This method is sensitive [7] but highly laborious. Furthermore, IBV may be detected directly in tissues of infected birds by means of immunohistochemistry [8,9] or *in situ *hybridization[10]. The reverse transcription-polymerase chain reaction (RT-PCR) has proved useful in the detection of several RNA viruses [11]. Aim of this study was first to evaluate the suitability of IHC for detection of IBV antigen in paraffin-wax embedded CAM and second to analyse the viral antigen distribution in different embryonic tissues between 48 and 120 h after experimental infection.

## Results

**Figure 1:**
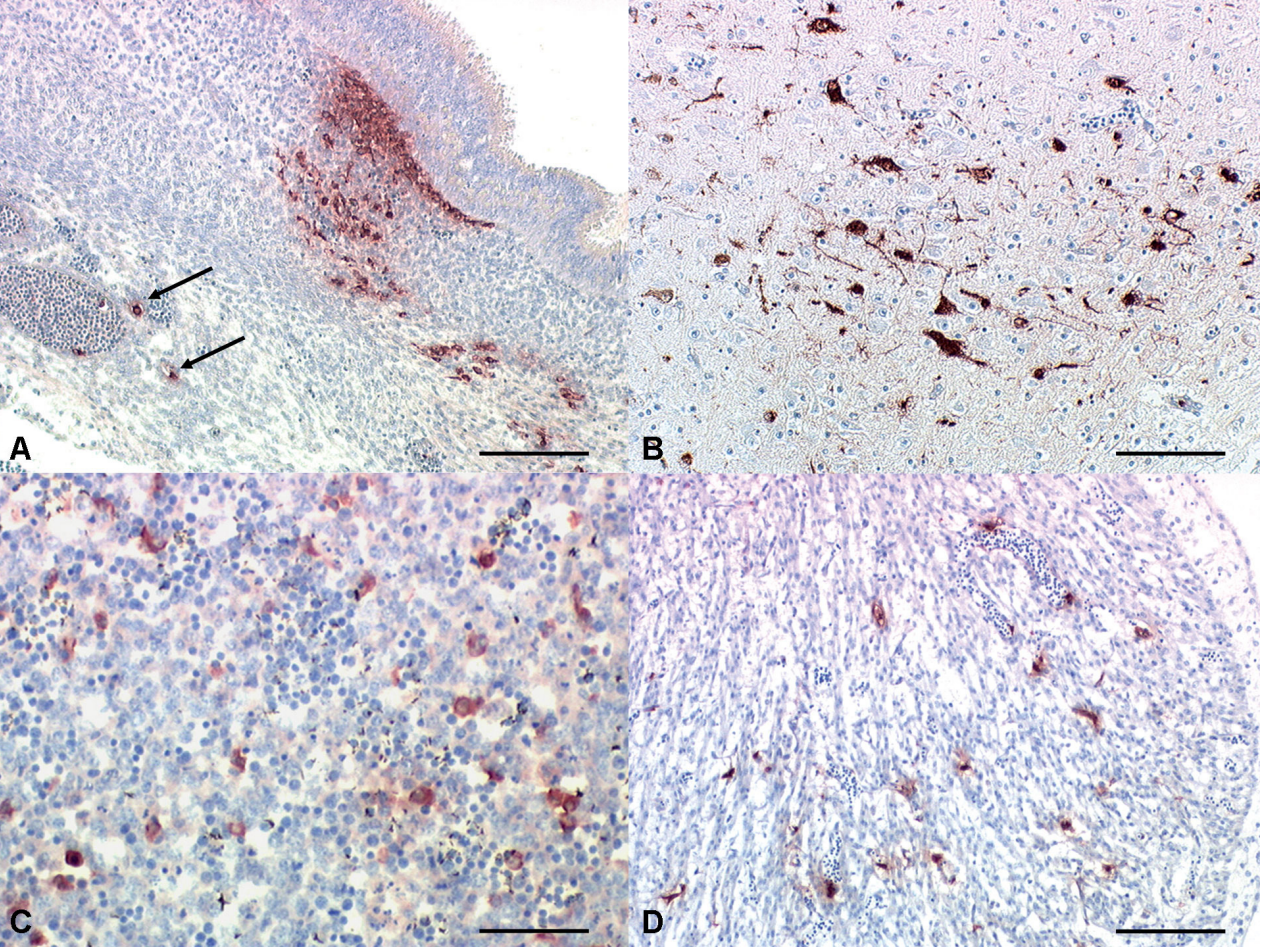
**Immunohistochemical detection of IBV antigens in chicken embryos after experimental infection with IBV M41**. (A) Proventriculus, focally extensive, there is strong immunolabelling of smooth muscle cells and scattered cells within the vasculature (arrows). Bar = 100 μm (B) Brain, within the cytoplasm of numerous neurons there is strong staining of IBV antigen. Bar = 40 μm (C) Spleen, scattered through the parenchyma there are numerous singular polygonal cells with red intracytoplasmic staining for IBV antigen, interpreted to be macrophages. Bar = 40 μm (D) Myocardium, capillary endothelium stains strongly positive for IBV antigen. Bar = 100 μm.

Inoculation of 10^3 ^EID_50 _IBV M41 in SPF ECE resulted in death of embryos at 24 h (3 embryos), 41 h (2 embryos) and single embryonic death at 65, 87 and 120 h after IBV inoculation. Allantoic fluid and CAM were harvested at the times given in Table 1 which also showed that high virus titers were obtained 24–87 h PI that decreased sharply to 10^2 ^TCID_50 _at 120 h PI (Table 1). Immunostaining of the CAM was positive in all inoculated eggs from which infectious virus was also recovered. Only one embryo showed non specific death at 24 h PI as it showed negative results in both immunostaining and virus recovery assays. IHC detected infected CAM that possesses virus titers of only 10^2 ^or 10^3 ^TCID_50 _in the corresponding allantoic fluid (Table 1). IBV antigens were detected in the nasal epithelium, trachea, lung, spleen, myocardium, liver, gizzard, proventriculus, kidney, skin, sclera of the eye, spinal cord, as well as in neurons of the central nervous system in infected embryos (Table 1, Figure 1). Moderate number of positive cells (++) were detected in the mucosa, smooth muscle fibres and vasculature of gizzard at 41, 48, 65 and 72 h PI. Few (+) to moderate (++) number of positive cells were detected in the proventriculus mucosa as well as its smooth muscle fibres in embryos dead at 41, 87 and 120 h PI (Figure 1A). Positive immunostaining was also detected in macrophages of both spleen (41, 48, 65, 72, 87 h PI) (Figure 1C) and liver (Kupffer cells) (41, 48, 65, 87 h PI). IBV antigens were also present in neurons of both spinal cord (65 and 72 h PI) and brain (65 and 120 h PI) (Figure 1B), heart (41, 48, 72 h PI) (Figure 1D), nasal cavity (48, 72, 87, 120 h PI), lung (41, 87 h PI), skin (48, 87, 120 h PI), eye sclera (65 h PI). In the kidney, viral proteins were detected in both renal tubules (72 h PI) and glomeruli (41, 48, 87 h PI) Table 1.

**Table 1:** Detection of IBV in CAM and chicken embryo.

Sample No.	Time after inocul ation (h PI)	Embryo status	Results of Immunohistochemistry	Virological findings‡ (TCID_50_/ml)
1	24	Dead	CAM -	0
2	24	Dead	CAM +	10^4.5^
3	24	Dead	CAM +	10^4.5^
4	41	Dead	CAM +	10^4^
5	41	Dead	CAM +	10^3^
			Gizzard (smooth muscle, vasculature) ++	
			Heart (vasculature) ++	
			Kidney (glomerular tufts) ++	
			Liver (Kupffer cells) ++	
			Lung (vasculature) ++	
			Proventriculus (smooth muscle) ++	
			Spleen (macrophages) +	
6	48	Alive	CAM +	10^4^
			Gizzard ++	
			Heart (vasculature) ++	
			Kidney (glomerular tufts) ++	
			Liver (Kupffer cells) +	
			Lung ++	
			Proventriculus +	
			Spleen (Macrophages) +++	
7	48	Alive	CAM +	10^6^
			Nasal mucosa (epithelium) ++	
			Skin (epidermis) +	
8	48	Alive	CAM +	10^5^
			Nasal mucosa (epithelium) ++	
			Skin (epidermis) +	
9	65	Dead	Brain (neurons) +	10^5^
			CAM: +	
			Eye (sclera) +	
			Gizzard ++	
			Liver +	
			Spinal cord (neurons) +	
			Spleen (Macrophages) ++	
10	72	Alive	CAM: +	10^6^
			Gizzard (mucosa) ++	
			Heart (vasculature)	
			Kidney (tubuli) +	
			Nasal cavity (mucosa) +++	
			Skin (epidermis) ++	
			Spinal cord (neurons) +	
			Spleen (macrophages) ++	
11	87	Dead	CAM +	10^5^
			Kidney (glomeruli) +	
			Liver (Kupffer cells) +	
			Lungs (epithelium, vasculature) +	
			Nasal cavity (mucosa)++	
			Proventriculus (smooth muscle, mucosa) ++	
			Skin (epidermis) ++	
			Spleen (macrophages) +++	
12	120	Dead	Brain (neurons) +	10^2^
			CAM +	
			Nasal cavity (mucosa) ++	
			Proventriculus (mucosa) +	
			Skin (epidermis) ++	
			Trachea (mucosa) ++	

Results of immunohistochemistry and virus reisolation.

Intensity of immunostaining: - (negative = no positive cells), + (weak = small number of positive cells per high power field), ++ (moderate = moderate number of positive cells per HPF), +++ (strong = accentuated staining pattern with large numbers of positive cells per HPF); CAM: chorioallantoic membrane.

‡Allantoic fluid from respective embryo was titrated in chicken embryo kidney cell culture and virus titter was expressed as TCID_50_.

## Discussion

Classic methods for IBV diagnosis include serological tests for analysis of antibody titers against IBV [12] and virus isolation in embryonated chicken eggs since IBV grows well in the developing chicken embryo. These methods are inherently slow and time consuming. Currently, detection and serotype analysis of IBV is performed by RT-PCR and restriction fragment length polymorphism analysis [13] or by sequencing RT-PCR products of S1 gene [14]. To establish IHC on paraffin-wax embedded tissues using a polyclonal rabbit anti-IBV serum, embryonated eggs were inoculated with 10^3 ^EID_50 _IBV M41. CAM and embryos were collected till 120 h PI, since the presence of IBV antigen in inoculated eggs by an antigen detection method is preferably performed 2 to 3 days after inoculation [15]. This also confirmed in the current study where IBV titers declined sharply at 120 h PI. It is well known that maximum IBV virus titers reached 1 to 2 days post-inoculation [16-18] but interestingly, allantoic fluid showed high virus titers 24–87 h PI. Immunostaining of the CAM was positive in all inoculated eggs from which infectious virus was recovered. It is worth to note that also samples of infected CAM with respective allantoic fluid virus titers of only 10^2 ^or 10^3 ^TCID_50 _were obtained, stained positively with the IBV antiserum which equals approximately 10^4 ^or 10^5 ^EID_50 _[19]. This indicates a relatively high sensitivity of the immunohistochemistry applied in this study. Using a monoclonal antibody for immunostaining, the detection limit for IBV antigen was 10^6.2 ^EID_50 _as described by [20]. These differences in sensitivity may be due to larger number of antigenic epitopes recognized by the polyclonal serum. This finding prompted us to apply IHC for screening of antigen distribution in different tissues in the IBV inoculated embryos. Antigens were detected in the nasal epithelium, trachea, lung, spleen, myocardium, liver, gizzard, proventriculus, kidney, skin, sclera of the eye, spinal cord, as well as in neurons of the central nervous system in infected embryos. However, isolation or detection of virus by virological methods may indicate only that virus is present in the tissue due to viraemia and thus does not necessarily prove productive replication in the respective organs. Hence, tissue tropism can not be determined by virus isolation or detection only [21] but requires morphologically based techniques. Although Chong & Apostolov [22], failed to detect virus by immunofluorescence in the intestine and cecal tonsils of chickens experimentally infected with M41 of IBV, Lucio & Fabricant [23] found that M41 can infect a variety of tissues and that some isolates may be recovered frequently from the digestive tract. IBV infection of the proventriculus was firstly recorded in China [3] then detected with UNAM-97 IBV Mexican variant that produced decrease in the proventricular gland papillary branching and electrodense material scattered in proventriculus with a structure consistent with coronaviruses [24]. To the best of our knowledge, this is the first time that the prototype IBV strain M41 was also detected within the muscle layer of the proventriculus. Because IBV causes an upper respiratory tract disease, viral antigens in nasal mucosa, trachea and lung were expected. IBV M41 viral antigen was found in the renal tubules and glomeruli. This observation is consistent with the finding that IBV M41 has also been isolated and/or detected in kidneys of naturally or experimentally inoculated chickens [23,25,26]. As earlier described for other IBV genotypes, antigens of M41 were present not only in renal tubules, but also in the glomerular tuft epithelium [27]. The detection of IBV antigen in the spleen, is discussed controversially in the literature. In some reports, IBV antigens or mRNA were not found within the spleen [28-30], in others, splenic infection was observed [27,31,32]. Studies to determine virus distribution in embryonic tissues were conducted previously [29,30] indicating that IBV antigen or nucleic acid was present in trachea, bursa, kidney, intestine, lung, heart, esophagus, mesentery, shell gland, and air sac, but not in spleen or thymus. The different virus distribution between the present paper and other papers may be due to the difference of embryo age at inoculation; 10 days old embryos (present paper) and 17 or 18 days old embryos [29,30]. It is probable that more immature tissues are more susceptible for IBV. The susceptibility for IBV infection is associated with the expression of 2,3-linked sialic acid which is used by the virus for primary attachment to the cell surface [5,6]. For tight binding and subsequent fusion with the cellular membrane interaction with a second receptor appears to be required [5]. The initial target of avian influenza viruses and IBV in chicken is the respiratory epithelium. Presence of 2,3-linked sialic acid is the prerequisite for avian influenza viruses to initiate respiratory infection. This molecule may also be used by IBV for infection of the respiratory tract. IBV, like avian influenza viruses, infects many non-respiratory tissues, including alimentary tract, oviduct and kidney [33,34]. The broad distribution of 2,3-linked sialic acid in different organs and species contradicts the view that this type of sugar is a major determinant of the narrow host tropism of IBV. In this study IBV antigen was found in musculature of both gizzard and proventriculus of inoculated embryos which is consistent with the known presence of 2,3-linked sialic acid receptors in the intestinal tract of chicken [35]. Thus it raises the question whether IBV infection of the proventriculus is a classic feature of IBV and occurs in association with an old IBV strain. A recently isolated M41 strain [26] resulted in an increased proventriculus index 7–28 days after experimental infection of 1-day-old chickens [Mohamed AA: Studies on infectious proventricultis in broiler chicken. Master thesis, In progress].

For our knowledge, IBV was neither isolated from nor detected in the brain of young or adult chickens [32]. Our finding that IBV antigens are present in the nervous system of embryonic chicken, may be explained by the presence of polysialylated N-CAM (neural cell adhesion molecules) in chicken embryos which might mediate virus entry into the neurons. The abundance of polysialylated N-CAM declines gradually during the embryonic development and the synthesis dramatically decreases right before birth [36,37].

## Conclusion

Our results show that the classic IBV strain M41 exhibits a wide tissue tropism including the nervous system and the proventriculus in chicken embryos and demonstrate that IHC as described here is a very sensitive tool for detection of productive virus replication in situ and therefore allows further studies to improve the understanding of the pathogenesis of the IBV infection.

## Methods

### Cells and viruses

The IBV strain M41 used in the current study was kindly provided by M. Hess, Intervet, Boxmeer, NL. Chicken embryo kidney cells (CEK) were prepared from specific pathogen free (SPF) embryonated chicken eggs (ECE) as described elsewhere [38]. Briefly, kidneys from 18-day-old SPF ECE were isolated, washed with Hanks' balanced salts solution, minced and disaggregated in trypsin solution. After centrifugation the cells were resuspended in Dulbeccos' modified Eagle's medium (DMEM) supplemented with 10% fetal calf serum (FCS) and grown at 37°C in a 5% CO_2 _incubator to confluent monolayers.

### Inoculation of embryonated eggs

Twelve ten days old SPF ECE were inoculated in the allantoic sac with 200 μl of IBV strain M41(10^3 ^EID_50_). Inoculated ECE were candled twice daily. At 48, 72, or 120 h dead and/or living whole embryos as well as allantoic fluid and CAM were collected. Embryos died at any time after inoculation, were also sampled accordingly. The allantoic fluid of inoculated eggs was harvested and clarified by centrifugation for 10 min at 925 g. The obtained supernatants were used for titration in CEK.

### Immunohistochemistry

The presence of IBV antigens was investigated in CAM and embryos using the avidin-biotin complex (ABC) method for immunohistochemistry [39]. To this end, sampled CAM and individual whole embryos, containing all organs were fixed for 4 h in Carnoy solution (absolute ethyl alcohol:chloroform:acetic acid; 6:3:1) followed by dehydration in absolute ethanol, and paraffin wax-embedding. Sections were cut at 2 μm, and mounted on electrostatically charged glass slides. Tissue sections (CAM and whole embryos cut in transversal or sagittal orientation) were dewaxed in xylene, rehydrated, treated with 3% hydrogen peroxide and then subjected to antigen retrieval by microwaving in 10 mM citric buffer (pH 6.0) for 10 min. Treated slides were cooled at room temperature for 20 min. Non specific background staining was blocked by incubating the sections for 20 min. with goat serum. The sections were then incubated for 1 h with 1:3000 rabbit anti-IBV polyclonal serum (prepared previously in the laboratory of G. Keil, FLI, using purified IBV M41) in Tris buffered saline (TBS), 1% bovine serum albumin (BSA), followed by 30 min. incubation with each of 1:200 biotinylated goat anti-rabbit immunoglobulin and subsequent extra avidin peroxidase conjugate. To visualize bound antibodies, the sections were then incubated in amino-ethyl-carbazole substrate chromogen (DakoCytomation, Hamburg, Germany) for 10 min. Finally, the sections were counter-stained with Mayer's haematoxylin and mounted with aqueous mounting medium. The staining intensities observed microscopically were divided into four grades: - (negative = no positive cells), + (weak = small number of positive cells per high power field [HPF, approx. 400×]), ++ (moderate = moderate number of positive cells per HPF), +++ (strong = accentuated staining pattern with large numbers of positive cells per HPF). In each tissue 10 randomly selected areas of each compartment were evaluated at high power by light microscopy. The judgments were made semiquantitatively via side-by-side comparisons of one section to another and the purpose was to evaluate antigen distribution in relation to labelling intensity in different embryonic tissues.

### Virus titration

100 μl of tenfold serial dilutions in MEM were added to 10^5 ^CEK cells per well. After 48 h of incubation, cells were fixed with acetone-methanol, and virus titers expressed as tissue culture infective dose fifty (TCID_50_) were determined by indirect immunofluorescence using a polyclonal serum raised in rabbits against purified IBV M41 virions.

### Indirect immunofluorescence

IBV infected CEK cells were fixed with acetone-methanol for 15 min. After washing, cells were incubated with the polyclonal rabbit anti-IBV serum (1:2000) for 1 h and subsequently with FITC-conjugated goat anti-rabbit antibodies (Sigma) (1:2000) for 1 h. Both primary and secondary antibodies were diluted in PBS. Plates were rinsed three times with PBS after each step.

## Competing interests

The authors declare that they have no competing interests.

## Authors' contributions

ASA performed virus inoculation in ECE and cell culture, IFA, contributed to IHC, analyzed the data, and drafted the manuscript. PZ contributed to IHC and histopathological examination, JV performed virus isolation and IFA. GMK and JPT initiated the project, provide continued directions and critically reviewed the manuscript. JPT also performed and supervised histopathology and IHC. All authors read and approved the final manuscript.
